# Sentinel Lymph Node Procedure in Pediatric Patients with Melanoma, Squamous Cell Carcinoma, or Sarcoma Using Near-Infrared Fluorescence Imaging with Indocyanine Green: A Feasibility Trial

**DOI:** 10.1245/s10434-022-12978-z

**Published:** 2023-01-15

**Authors:** Bernadette Jeremiasse, Cecilia E. J. Terwisscha van Scheltinga, Ludwig E. Smeele, Nelleke Tolboom, Marc H. W. A. Wijnen, Alida F. W. van der Steeg

**Affiliations:** 1 Princess Maxima Center for Pediatric Oncology, Heidelberglaan 25, 3584 CS Utrecht, The Netherlands; 2 Antoni van Leeuwenhoek, Plesmanlaan 121, 1066 CX, Amsterdam, The Netherlands; 3 Amsterdam UMC, Meibergdreef 9, 1105 AZ Amsterdam, The Netherlands; 4 Division Imaging and Oncology, Department of Radiology and Nuclear Medicine, University Medical Center Utrecht, Heidelberglaan 100, 3584 CX Utrecht, The Netherlands

## Abstract

**Background:**

Standard sentinel lymph node procedure (SNP) in pediatric cancer consists of a preoperative injection with ^99m^technetium nanocolloid in combination with an optional intraoperative injection with blue dye. However, blue dye has disadvantages, and the detection rate is low, with only 60% of sentinel lymph nodes (SLNs) staining blue. In adult oncology, fluorescence imaging using indocyanine green (ICG) has been shown to be a safe and accurate method for visual detection of SLNs, with a higher sensitivity (up to 97%) compared with blue dye. Therefore, our aim is to determine the feasibility of the addition of ICG to ^99m^technetium nanocolloid (ICG–TC) for visual detection of SLN in pediatric patients.

**Methods:**

A total of 15 pediatric patients with melanoma, squamous cell carcinoma, and sarcoma were prospectively included. Preoperatively, patients were injected with ICG–TC and imaging with lymphoscintigraphy and single-photon emission computed tomography– computed tomography was performed. Intraoperatively, SLN was detected with fluorescence and the gamma probe. Postoperatively, fluorescence was quantified by tumor-to-background ratio (TBR) and surgeons evaluated the use of ICG using a standardized questionnaire.

**Results:**

In 10/15 (67%) patients, SLNs were visible transcutaneously. Of all intraoperatively detected SLNs, 35/37 (95%) were fluorescent and 37/37 (100%) were radioactive. Furthermore, ICG–TC led to the identification of six additional SLNs as compared with preoperative imaging. The median TBR in vivo was 6.5 (IQR 5.3). The surgical evaluation showed that ICG assisted in SLN detection and was easy to use.

**Conclusions:**

ICG–TC for the SNP is a feasible procedure in pediatric patients. It showed an accurate detection rate, was helpful for visual guidance, and no adverse events occurred.

The standard-of-care sentinel lymph node (SLN) procedure in pediatric patients with cancer consists of a preoperative intradermal or peritumoral injection with ^99m^technetium nanocolloid as radiotracer in combination with an intraoperative injection of blue dye for visual guidance. This method has been proven to accurately detect SLN in pediatric patients with melanoma and sarcoma, with a detection rate of around 95%.^1,2^

Successful SNP depends on SLN localization using a dye and/or radiotracer, such as ^99m^technetium-nanocolloid, that can be visualized with preoperative imaging and detected intraoperatively by a handheld gamma probe. However, disadvantages of using a radiotracer are the potential disturbance of the radioactive signal originating from the injection site, a low spatial resolution, and the fact that the surgery has to be interrupted to use the gamma probe. Consequently, detection of SLN is sometimes complicated and demanding. The radiotracer limitations can be eliminated by using an agent that could aid visual guidance simultaneously without interrupting the surgery. Therefore, when visual guidance is wanted by the surgeon, the use of blue dye is currently added to the SLN procedure. However, blue dye has evident disadvantages, such as risk of (severe) allergic reactions (2%^3^) and long-lasting tattooing (41% after 12 months^4^), in addition to the limited penetration depth and alteration of the surgical field.^3–5^ Furthermore, the efficacy is low, with only 60% of SLNs staining blue.^6^

In adult patients with melanoma, near-infrared (NIR) fluorescence imaging using indocyanine green (ICG) has been shown to be a safe, efficient, and accurate method for intraoperative visual detection of SLNs, with a higher sensitivity (up to 97%) compared with blue dye.^6^ However, in most studies ICG is used in combination with ^99m^ technetium nanocolloid, potentially overestimating the sensitivity of ICG alone. For pediatric patients, good safety profiles for the use of ICG have been established for indications other than SLN.^7^

Our aim is to determine the feasibility of the addition of ICG to ^99m^technetium nanocolloid for visual detection of SLN in pediatric patients. Owing to the high sensitivity of ICG in adults and the good safety and favorable side effects profile of ICG compared with blue dye, we expect that a combined injection of ICG noncovalently bound to ^99m^Tc nanocolloid (ICG–^99m^ Tc nanocolloid, ICG–TC) is able to improve visual guidance during surgery, while preventing the considerable risk of side effects associated with blue dye.^8^

## Patients and Methods

### Patients

This study (NL71166.041.20) was approved by the Institutional Review Board.

Inclusion criteria were: 1. Under the age of 18 years; 2. Diagnosis of melanoma, sarcoma or squamous cell carcinoma of extremity, head and neck, or trunk; and 3. Indication to undergo a SLN procedure. Exclusion criteria were: 1. Allergy to iodine; 2. Hypersensitivity to ICG; 3. Kidney insufficiency (eGFR < 45); and 4. Clinically manifest hyperthyroidism or autonomous thyroid adenoma. In total, 15 patients, (9 with melanoma, 4 with sarcoma, and 2 with squamous cell carcinoma) were prospectively included after giving informed consent (Table 1). All patients were clinically node negative as assessed by palpation.

**Table 1:** Characteristics of 15 pediatric patients that underwent a sentinel node procedure with ICG combined with ^99m^ technetium nanocolloid, including the number of sentinel lymph nodes (SLNs) identified preoperatively and intraoperatively with the different modalities: indocyanine green (ICG) and Technetium (Tc)

Paliant	Sex (M/R	Ago (years)	Indention	Tumor location	1 or 2 day protocol	SLNs preoperatively: nr (location)	SLN transcutaneous visible	SLNs identified with LCG: nr (location)	SLNs identified with Tc: nr (location)	Pathology SLNs
1	M	14	Melanoma	Lower arm, right	2-day	3 (elbow, 2 axilla)	NA	2 (1 elbow, 1 axilla)	2 (1 elbow, 1 axilla)	No metastasis
2	M	14	Squamous cell carcinoma	Maxilla, left	1-day	3 (2 left cervical, 1 right cervical)	No	3 (2 left cervical, 1 right cervical)	3 (2 left cervical, 1 right cervical)	No metastasis
3	F	11	Melanoma	Foot right	1-day	1 (inguinal)	Yes	2 (inguinal)	2 (inguinal)	No metastasis
4	M	8	Melanoma	Foot, right dig 3	1-day	3 (2 inguinal, 1 popliteal)	No	2 (2 inguinal, popliteal exduded)	2 (popliteal exduded)	1 SLN < 0.1mm
5	M	8	Squamous call carcinoma	Maxilla, right	2-day	3 (2 right cervical, 1 left cervical)	Yes	2 (right cervical)	3 (2 right cervical, 1 left cervical)	No metastasis
6	F	6	Melanoma	Hairy scalp	1-day	2 (1 dorsal right ear, 1 right cervical level 2B)	Yes	3 (2 dorsal right ear, 1 right cervical)	3 (2 dorsal right ear, 1 right cervical)	No metastasis
7	F	0	Rhabdomyosarcoma	Upper leg, right	1-day	2 (inguinal)	Yes	4 (inguinal)	4 (inguinal)	No metastasis
8	M	6	Synovial sarcoma	Foot, right	1-day	2(1 poplitea,1 inguinal)	partly (knee no, inguinal yes)	2 (1 poplitea, 1 inguinal)	2 (1 poplltea.1 inguinal)	No metastasis
9	M	6	Melanoma	Cheek right	1-day	2 (level lb right cervical, level 2 right cervical)	Yes	2 (level lb right cervical, level 2 right cervlcal, level 2 right cervical)	2 (level lb right cervical, level 2 right cervical)	No metastasis
10	F	10	Rhabdomyosarcoma	Foot, left	1-day	3 (2 inguinal, 1 popliteal)	Yes	3 (inguinal, popliteal exduded)	3 (3 inguinal, popliteal exduded)	1 SLN containing a metastasis
11	F	13	Melanoma	Back	1-day	3 (1 inguinal, 2 flanks)	partly (left flank yes, rest nor)	2(1 inguinal, 1 (flank)*	3 (1 inguinal, 2 flanks)	No metastasis
12	F	8	Melanoma	Shoulder, left	1-day	2 (axilla left, 1 left cervical level 5)	Yes	1 (axilla, cervical exduded)	1 (axilla, cervical excluded)	1 SLN containing a metastasis
13	F	11	Rhabdomyosarcoma	Lower arm, right	1-day	1 (axilla right)	No	2 (2 axilla right)	2 (2 axilla right)	No metastasis
14	F	13	Melanoma	Shoulder, left	1-day	3 (2 axilla left, 1 axilla right)	No	3(2 axilla left, 1 axilla right)	3 (2 axilla left, 1 axilla right)	No metastasis
15	F	8	Melanoma	Lower leg, left	1-day	2 (inguinal)	Yes	2 (inguinal)	2 (inguinal)	2 SLNs containing a micnxiietastasis (< 1mm)

*NA* not assessed

^*^In this case, 1SLN in the flank was fluorescent both in vivo and ex vivo, and the other flank, SLN, was only fluorescent ex vivo

### Tracer Preparation, Administration, and Preoperative Imaging

Preparation and labeling of ICG–TC was performed in the UMC Utrecht according to the good manufacturing practice (GMP) guidelines. Patients received a preoperative injection of either 100 MBq ^99m^technetium nanocolloid on the day of surgery (4–6 h before surgery, 1-day protocol), or 240 MBq the day before surgery (20–24 h before surgery, 2-day protocol) premixed with 0.25 mg ICG (ICG Pulsion, Pulsion Medical Systems, Munich, Germany) The injection was given in four intradermal deposits around the scar tissue of the primary tumor in patients with melanoma and peritumoral in patients with sarcoma and squamous cell carcinoma. Afterward, dynamic and early plus late static lymphoscintigraphy, and in most cases SPECT–CT images, were acquired to identify the locations of SLNs on a Symbia Intevo 16 Bold SPECT scanner (Siemens Healthineers, Erlangen, Germany). SLN was defined as the first lymph nodes on a direct lymphatic drainage pathway from the injection site. It is important to note that this is not always just one SLN. The tumor could drain on several SLNs at the same time. The location of the SLN was verified using a handheld probe and marked on the skin using a non-fluorescent marker.

### Surgical Procedure

Preoperatively, the near-infrared (NIR) fluorescence camera (Quest Spectrum 2.0, Quest Medical Imaging, the Netherlands) was used to investigate whether the SLN was detectable transcutaneously. During the operation, detection of SLNs was first assessed using a near-infrared (NIR) fluorescence camera, after which the handheld gamma probe (EuroProbe 3.2; PI-Medical, the Netherlands) was used for radioactive detection of SLNs. All lights in the operating room were dimmed and windows closed when acquiring the fluorescent images to reduce background signal. NIR images were taken with different exposure times (auto exposure, 25 ms, 50 ms, 100 ms) to capture the most optimal TBR. The wound bed was controlled for residual fluorescent or radioactive signal after SLN excision according to the same procedure as described for SLN detection. If residual signal was present, the surgeon searched for a second SLN. If no SLN could be detected by use of the gamma probe and NIR fluorescence camera, the surgeon could opt for the additional use of a peritumoral injection with blue dye.

### Postoperative Analysis

NIR fluorescence and radioactivity of the excised lymph nodes was confirmed on the back table in a black box using the same detection systems as during the operation. Fluorescence was quantified by tumor-to-background ratio (TBR) using Quest Spectrum TBR tool and surgeons evaluated the use of ICG using a standardized questionnaire (Table 2).

**Table 2:** Surgeon questionnaire; answer possibilities: 1 fully disagree, 2 disagree, 3 agree, 4 fully agree

Question	Average answer
Fluorescence helped me detecting the SLN	3.1
I found an extra SLN with the use of fluorescence	1.7
Fluorescence was easy in use	3.3
I found it hard to work with both fluorescence and the gamma probe	2.1
I found it hard to work with a screen during an open procedure	1.7
I prefer fluorescence/blue dye	14/15 fluorescence
I expect less complications due to the use of fluorescence	2.3
Patients and parents understood the SNP using fluorescence	3.4
I would recommend fluorescence for the SNP to patients	3.6
I would recommend fluorescence for the SNP to colleagues	3.5

*SLN*: sentinel lymph node, *SNP*: sentinel node procedure

### Pathological Evaluation

The resected lymph nodes were routinely analyzed by histopathological frozen section analysis. SLNs were fixed and embedded in formalin and paraffin for hematoxylin, eosin, and immunopathological staining at multiple levels according to the national protocol.

## Results

### Pre- and Intraoperative Results

In 10/15 patients (67%), some or all SLNs were already visible transcutaneously (Table 1). A total of 35 SLNs were identified on preoperative imaging, including both lymphoscintigraphy and SPECT–CT. In three patients, SLNs were identified in two nodal basins, and it was decided to only remove the inguinal and axillary SLNs since only these would influence adjuvant treatment decisions. Thus, 32 out of these 35 preoperatively identified SLNs were intraoperatively assessed. A total of 29/32 (91%) were detected with fluorescence, while 31/32 (97%) could be detected on the basis of radioactivity. One SLN could not be identified intraoperatively. SLNs that were only detected with radioactivity had a low fluorescent signal as measured ex vivo in the black box, which was not sufficient to be visible against the background in vivo. However, ex vivo these SLNs were mildly or clearly fluorescent (TBR ex vivo 3.4 and 27.4, respectively). In one patient with one SLN in the elbow and two in the axilla on preoperative imaging, only two SLN could be found, one in the elbow and one in the axilla.

Next to the 31/35 preoperatively identified SLNs that were detected during surgery, 6 additional SLNs in five patients were identified (all were both fluorescent and radioactive). The additional six SLNs were probably not detected preoperatively since they were in close contact with each other. For patients three and six, the additional SLNs were already visible transcutaneoulsy with ICG. The additional SLNs of patients six and seven were close to the tumor, which complicated pre- and intraoperative radioactive detection because of the high signal in the injection site. This led to a total of 37 intraoperatively detected SLNs. Of these, 35 (95%) were fluorescent and 37 (100%) were radioactive. Importantly, no adverse events occurred.

### Histopathological Findings and Postoperative Analyses

Two patients (no. 10 and 12) were diagnosed with metastases in their SLN. Two patients (no. 4 and 15) had micrometastases.

We started with both a 1 or 2 day protocol for tracer injection, based on the standard-of-care ^99m^technetium nanocolloid injections. Initially, we did not expect any differences between these protocols, but after two 1-day cases, we felt that fluorescence was more apparent when using this protocol. Therefore, we performed the remaining procedures with the 1-day protocol.

**Fig. 1:**
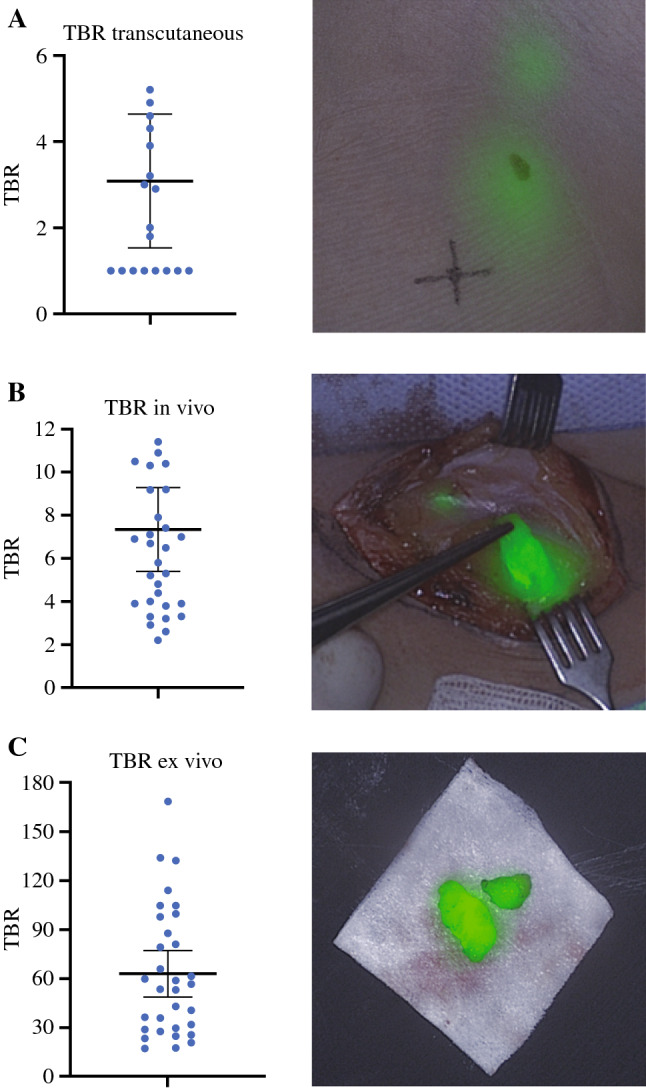
Graphs representing the individual tumor-to-backgorund ratios (TBR) of the sentinel lymph nodes (SLNs) and their respective mean and standard deviation in three different circumstances: (A) transcutaneous, (B) in vivo, and (C) ex vivo

All excised SLNs were fluorescent ex vivo. Ex vivo analysis showed a median TBR of 54.9 [interquartile range (IQR) 60.9; Fig. 1]. The median in vivo TBR was 6.5 (IQR 5.3) and the transcutaneous median TBR 2.0 (IQR 3.1). Images of the same SLN were made with four different exposure times. This led to four TBRs for the same SLN. In all cases, the most optimal TBR was selected for postoperative analysis. This TBR was usually based on the image generated with an exposure time of 25 ms. During surgery, the exposure time that revealed the best intraoperative image was used, which was usually the autoexposure (38 msec).

The surgical evaluation showed that ICG assisted in SLN detection, although its extent was dependent on the anatomical location of SLN. Surgeons evaluated ICG as easy to use and preferred it over blue dye. They would recommend ICG for the SNP to their colleagues and patients (Table 2).

## Discussion

We showed that ICG–TC can be used for visual detection of SLN in pediatric patients. It showed an accurate detection rate of 100%, meaning that in every patient at least one SLN was detected with fluorescence. Furthermore, 91% of the preoperatively detected SLNs and 95% of the intraoperatively detected SLNs was fluorescent. Finally, no adverse events occurred.

This is the first time that the use of ICG has been investigated for the SNP in pediatric patients. Our results in terms of detection rate and occurrence of adverse events are in line with the results in adult studies.^6,9–16^ These studies concerned adult patients with melanoma, but the SNP can also be of use in pediatric patients with sarcoma and squamous cell carcinoma.^1,17^ Our identified transcutaneous detection rate of 67% was higher compared with adult studies that ranged between 21 and 63%,^10,14,15^ most likely due to the amount of subcutaneous adipose tissue.^14^ Some adult studies also compared the use of ICG as visual tracer with blue dye. They all identified a higher detection rate of ICG.^6,9–13,15^ We did not compare ICG directly with blue dye. Nevertheless, we gave the surgeons the option to use blue dye if they expected ICG to be insufficient, which proved to be unnecessary.

The current study method was designed to investigate the feasibility of using ICG for visual detection of SLN in addition to ^99m^technetium nanocolloid in pediatric patients. ICG was therefore coupled with ^99m^technetium nanocolloid, resulting in the fact that we cannot draw conclusions on ICG or ^99m^technetium nanocolloid alone. Nonetheless, we chose this study design because we believe that ICG can be of additive value to a radiocolloid, but is not its replacement.^15,18–20^ ^99m^technetium nanocolloid is known to stain the SLN, so an advantage of ICG coupled with ^99m^technetium nanocolloid is that they both stain the SLN. Uncoupled ICG, however, travels easily to subsequent lymph nodes, increasing the chance of missing the SLN and unnecessary removal of non-SLNs.^14,21^ Perfect timing of the LN visualization is therefore crucial when ICG alone is used. On the other side, a potential downside of ^99m^technetium nanocolloid, and therefore also of ICG–TC, is that it might not be able to stain lymphatics and SLNs that are obstructed by tumor because of its molecular size.^22,23^ However, obviously involved nodes on preoperative imaging are often a contraindication to performing a SNP, or they are resected in addition to the SNP.

On the basis of this feasibility study, we expect that ICG as addition to ^99m^technetium nanocolloid could potentially lead to an improved detection rate, which could reduce false negatives, thereby further improving the diagnostic value of the SNP. The systematic review of Lafreniere et al. showed that ICG contributed to the identification of 2% of the total number of SLNs harvested.^19^ We noted that in five cases, the use of ICG led to the identification of six additional SLNs in total that would not have been identified with ^99m^technetium nanocolloid alone because they were either in close proximity to each other or to the injection site, causing disturbance of the radioactive signal. Furthermore, three of our four cases with metastatic disease had SLNs that were both detected preoperatively and intraoperatively. In the fourth case, an additional SLN was found next to the two preoperatively identified SLNs. However, we do not know whether the metastatic SLN was seen on the preoperative images.

The additive value of ICG for the SNP will also depend on the anatomical location of the SLN. We noted that ICG is more of value in the inguinal area and in transit sites compared with the axilla, probably due to the amount of adipose tissue covering the SLN in combination with the maximum depth penetration of ICG. This was also noted by Namikawa et al., who showed that a higher BMI and the axillary node field were factors predicting SLN detection failure.^14^ In addition, the head and neck area, which is known to be a difficult area for SLN harvesting, may benefit relatively more from ICG.^19,20,24^ SLNs in this area are often bilateral, located in multiple LN basins and with many LNs clustered together.^25,26^ Additionally, the primary tumor is often in close proximity, disturbing the radioactive signal. Nevertheless, the advantage of ^99m^technetium nanocolloid is the preoperative SLN localization, which may be bilateral. Therefore, a combination of ICG–TC again seems to be most optimal.

Next to improving the detection rate, ICG could, in cases of transcutaneous visibility, allow for a smaller incision and a more selective dissection with limited disruption of the lymphatics. This has the potential to reduce morbidity at the operation site, which was already shown by Korn et al., who needed less drains postoperatively.^13^ Additionally, the improved SLN visualization by use of ICG might reduce operating time,^13^ but this is probably also dependent on the anatomical location of the SLN. In this study we have not measured operating time. ICG could also potentially improve the flow of the surgery, since the camera could be placed in a position that allows for continuous visualization. However, in this situation only the camera’s light source can be used for surgery, as other light sources in the operating theater contain NIR light that disturbs the fluorescence signal.

Introducing a novel tool in the operation room is only valuable if surgeons are convinced of the added value. We used questionnaires that gave insight into the perspective of the surgeons on fluorescence for the SNP. Overall, they were positive with regard to the visual guidance of ICG in SLN detection and they would recommend the use of ICG to their colleagues and patients. The answers given in the questionnaire, as well as the SLN detection, could have been influenced by the fact that adaptation to a novel technique and learning curve could differ per surgeon. In this study, the use of ICG for SLN detection was new for four out of five surgeons. Nevertheless, all surgeons preferred the use of ICG over blue dye and they were able to identify fluorescent SLNs in all cases.

It should be clear that this study concerns small patient numbers, as it is a feasibility study. However, our identified detection rate of ICG-TC is promising and in line with detection rates of ^99m^technetium nanocolloid reported in literature. Currently, we cannot conclude whether this method improves the diagnostic accuracy of the SNP, and/or influences the surgery time, complication rate, and facilitation of the operation. There are also some general limitations when using fluorescence. First of all, it might be necessary to disturb the surgical procedure when the lights have to be turned off and the camera positioned. As already mentioned, fluorescence has a maximum depth penetration leading to reduced utility for deeper-lying SLNs. Finally, ICG cannot be used to localize SLN with preoperative imaging.

Altoghether, we expect that ICG–TC is the best tracer to use for the SNP. Larger, well-designed studies are needed to investigate the implications of fluorescence as an addition to ^99m^technetium nanocolloid on the SNP diagnostic accuracy by measuring the false negative rates. Its implications for facilitating surgery, reducing surgery time, and reducing the number of complications should also be explored.

However, there are institutes in which radioactive tracers are not available. In these institutes, blue dyes are most commonly used. Adult studies indicate that ICG alone performs better than blue dye,^6,9–13,15^ so a well-designed study to compare the use of ICG with blue dye in pediatric patients is needed. It can be hypothesized that ICG performs even better in this patient population since skin thickness and average BMI of pediatric patients is often lower compared with adult patients. Yet, for these institutes it might be wise to combine ICG with blue dye in cases with a high BMI and/or axillary node field, since these are factors predicting SLN detection failure with ICG. This should be decided preoperatively, as the lymphatics may have been disrupted if the procedure has already started.

In conclusion, ICG–TC for the SNP is a feasible procedure in pediatric patients. It showed an accurate detection rate, was helpful for visual guidance, and did not lead to tattooing or any other adverse events.
